# The genetic change in *P. falciparum* populations of rural Tanzania resulting from national policy on firstline malaria treatment and pilot Sulfadoxine/pyrimethamine and Artesunate combination

**DOI:** 10.1186/1475-2875-9-S2-P20

**Published:** 2010-10-20

**Authors:** Allen Malisa, Richard Pearce, Salim Abdullah, Hassan Mshinda, Patrick Kachur, Peter Bloland, Cally Roper

**Affiliations:** 1 Ifakara Health Institute, Box 53, Ifakara, Tanzania; 2 Sokoine University of Agriculture, Department of Biological Sciences, PO Box 3038, Morgoro, Tanzania; 3 London School of Hygiene and Tropical Medicine, Keppel Street, London, WC1E7HT, UK; 4 Malaria Epidemiology Branch, Centers for Disease Control and Prevention, Atlanta, GA, USA

## 

Theory predicts that we can protect the efficacy of future antimalarials by changing treatment practice or drug formulation, but the potential success of such interventions rests upon their impact on drug pressure in the field. So far, gathering field data on the relationship between policy, drug pressure, recombination and the evolution of resistance has been entirely challenging. To test these predictions, *dhfr and dhps frequency* changes were measured in two rural districts of Rufiji and Kilombero/Ulanga during 2000-2006, and the frequencies of the two genes compared prior, during and after antimalarial policy change from first line CQ to first line SP in 2001. Furthermore, while SP first line was maintained in Kilombero/Ulanga, pilot combination therapy of SP+Artesunate (ART) was introduced in Rufiji in 2002 to replace SP and *dhfr* and *dhps* frequency changes compared between the two districts. Size polymorphisms at three sets of microsatellite loci linked to *dhfr* and three other sets of unlinked microsatellite loci were analysed. Genetic analysis of SP resistance genes was carried out on 9,662 *Plasmodium falciparum* infections identified in a series of annual cross sectional surveys conducted in the two districts between 2000-2006.

The frequency of *dhfr* and *dhps* resistance alleles did not change significantly while SP was the recommended second-line treatment, but highly significant changes occurred during the subsequent year after the switch to first line SP. The frequency of the triple mutant *dhfr* allele increased by 37% -63% and that of double mutant *dhps* allele increased 200%-300%. A strong association between these unlinked alleles also emerged; confirming that they are co-selected by SP. Distribution of major lineages indicates that there is extensive genetic exchange among the geographic regions. Combination therapy had visible effect on the frequencies of *dhfr* and *dhps* resistance alleles. The findings of this study provide insight on the interplay between policy, drug pressure, recombination and the evolution of resistance.

